# A longitudinal mediation study of peer victimization and resting-state functional connectivity as predictors of development of adolescent psychopathology

**DOI:** 10.3389/fpsyt.2023.1099772

**Published:** 2023-03-23

**Authors:** Hanie Edalati, Mohammad H. Afzali, Sean Spinney, Josiane Bourque, Alain Dagher, Patricia J. Conrod

**Affiliations:** ^1^CHU Sainte-Justine Research Center, University of Montreal, Montreal, QC, Canada; ^2^Department of Psychiatry, Perelman School of Medicine, University of Pennsylvania, Philadelphia, PA, United States; ^3^Montreal Neurological Institute, McGill University Health Centre, Montreal, QC, Canada

**Keywords:** peer victimization, resting-state functional connectivity, developmental psychopathology, social pain, adolescence

## Abstract

**Background:**

Peer victimization (PV) is associated with alterations in neural responses in regions subserving emotional regulatory processes and with increased risk of psychopathology during adolescence. The present study examined the longitudinal mediating effects of resting-state functional connectivity (rsFC) between adolescent PV and subsequent internalizing (depression and anxiety), and externalizing (conduct and hyperactivity/inattention) symptoms.

**Methods:**

151 adolescents (baseline mean age 12–14; 54% males) were assessed and imaged three times during a five-year period. We focused on rsFC of *a priori* determined Regions-of-Interest (ROIs) guided by the literature (i.e., amygdala, anterior and posterior insula, anterior cingulate cortex, and medial prefrontal cortex). Multilevel mediation (MLM) analyses simultaneously examined the between-person, concurrent within-person, and lagged within-person associations between PV and internalizing/externalizing symptoms through changes in couplings of the amygdala with the other four ROIs. All models controlled for the effects of self-reported childhood maltreatment and sex differences.

**Results:**

An increased rsFC of the amygdala-posterior insula significantly mediated the lagged within-person association of PV and internalizing symptoms (β = 0.144; 95% CI [0.018, 0.332]). This effect was significant regardless of childhood maltreatment, concurrent externalizing symptoms, and sex differences. The rsFC did not mediate the relationship between PV and externalizing symptoms.

**Conclusions:**

Results of this study suggest that adolescent PV may lead to long-lasting maladaptive neural communication between emotional response and sensory perception of pain (i.e., bottom-up emotion regulation) and that these neural responses may serve as unique markers for increased internalizing symptoms that appear in later adolescence in peer-victimized youth. These findings have implications for interventions targeting internalizing symptoms in victimized adolescents.

## Introduction

Adolescence is a developmental period in which peer’s approval and evaluation becomes central to the individual’s development of self-concept and interpersonal experiences (1). Exposure to peer victimization (PV), such as the experience of targeted physical and relational aggression, social exclusion or systematic rejection by the peer group, is a strong risk factor for the development of mental health disorders in adolescents (2–4). Previous research has reported that peer-victimized adolescents present with more difficulties in regulation of emotions and affective response in the context of potential social exclusion and rejection which, in turn, increases their vulnerability to psychopathology (5, 6). For example, they are more likely to show biased appraisals in the context of conflicts with peers (7), and attribute hostile intent to peers in hypothetical provocation situations (8, 9). However, little is known about the neural correlates explaining this path in the association between adolescent PV and psychopathology.

Evidence from studies using functional magnetic resonance imaging (fMRI) to examine the neural response to experimental manipulation of social exclusion suggests the involvement of a set of regions that exist across brain networks implicated in the affective and sensory processing of emotions, namely amygdala, and subregions of anterior cingulate cortex (ACC) and insula (6, 10–12). The subgenual ACC (sgACC) and anterior insula (implicated in the affective processing of social pain and rejection) as well as posterior insula (implicated in the sensory processing of pain), are particularly involved in the self-reported distress in response to social pain and exclusion in adolescents (13). The ACC and dorsal anterior insula are also part of the salience network that modulates switching between the default mode network (DMN) and the central executive network, and facilitates access to cognitive resources including attention and working memory in response to emotional and salient stimuli (14, 15). In addition to the amygdala, ACC, and anterior and posterior insula, alterations in the medial prefrontal cortex (mPFC) have been shown to play a role in vulnerability to psychopathology in peer-victimized adolescents [e.g., (12, 16)]. The mPFC is part of the default mode network (DMN) involved in emotional self-reference processing, such as top-down processing of one’s own emotions and self-awareness processes (17, 18).

Research on adolescents with chronic experience of PV has indicated a greater activation in amygdala and dorsal ACC (dACC) during social exclusion relative to inclusion in victimized vs. non-victimized adolescent girls (6). This study compared 24 adolescent girls with histories of chronic PV with 23 non-victimized girls on their neural response to social exclusion and internalizing symptoms (i.e., depressive symptoms and social anxiety). Findings showed that greater activation of dACC, sgACC, and anterior insula in response to social exclusion was associated with reporting greater internalizing symptoms. This association was stronger in chronically victimized compared to non-victimized girls. In addition, avoidance motivation (i.e., “psychological sensitivity to social punishment”) mediated the association between neural sensitivity in these three regions and internalizing symptoms in peer-victimized adolescents (6). These findings suggest that victimized relative to non-victimized girls are more vigilant and emotionally reactive to the experience of peer rejection and exclusion, which is associated with a greater risk of internalizing and affective disorders. However, the longitudinal direction of the effects of PV on these neural processing and risk of internalizing symptoms, and/or the experiences of peer-victimized boys and their mental health outcomes is less clear.

Recent studies have investigated the functional connectivity or temporal correlation in the activity of this cross-network of regions involved in emotional regulation to better understand neural correlates of psychopathology in victimized youth. There is evidence that alteration in the functional connectivity of the amygdala with these other regions (i.e., ACC, insula, and mPFC) is associated with difficulties in emotion processing and regulation (19–21), and increased risk of both internalizing and externalizing symptoms (22–25). A recent cross-sectional study has examined the changes in functional connectivity of the left amygdala, mPFC, ACC, and right insula (i.e., fronto-limbic network) during a social inclusion/exclusion task among peer-victimized, defenders (i.e., those with histories of defending peers), and non-victimized adolescents (*n* = 15 each group) (12) and how it relates to the risk of internalizing symptoms (12). Findings from this study indicated that peer-victimized adolescents show significantly weaker, negative functional connectivity between the left amygdala-ACC and left amygdala-right insula in continuous functional connectivity across inclusion and exclusion conditions and connectivity within inclusion condition compared to the controls. This study also indicated that the positive functional connectivity of the left amygdala-mPFC across inclusion and exclusion moderates the association between prior PV and depressive symptoms (12). The altered patterns of connectivity between the amygdala and important regions involved in affective processing of social pain (ACC and insula), and emotional self-reference processing (mPFC) reflect on the negative impact of PV on emotional regulatory processes in response to social exclusion and internalizing problems in peer-victimized adolescents. Although this cross-sectional study provided unique findings about functional connectivity of network of regions implicated in emotional response to social exclusion in peer-victimized adolescents, the temporal precedence from PV to these neural processes and subsequent psychopathology is less clear.

The present study aimed to address this gap by examining the resting-state functional connectivity (rsFC) of these regions in relation to experiences of peer victimization. The rsFC quantifies the intrinsic activity and functional connections of specific brain regions at rest in the absence of any explicit task and is able to identify more long-lasting and global abnormal communications in neural networks. Guided by findings of previous studies on regions implicated in the emotional regulatory processes in peer-victimized adolescents (6, 12), we investigated rsFC of a network of *a priori* determined Regions-of-Interest (ROIs) including the amygdala, ACC, anterior and posterior insula, and mPFC. We examined the mediating effects of the amygdala connectivity with ACC and anterior and posterior insula, and mPFC in the relationship between adolescent PV and subsequent psychopathology symptoms using a longitudinal design over a period of five years. This study also uniquely investigates the role of functional connectivity in the association between PV and adolescent’s vulnerability to both internalizing and externalizing symptoms. There is evidence for a relationship between alterations in the functional connectivity of these regions and increased risk of externalizing problems (19, 24, 25).

**FIGURE 1:**
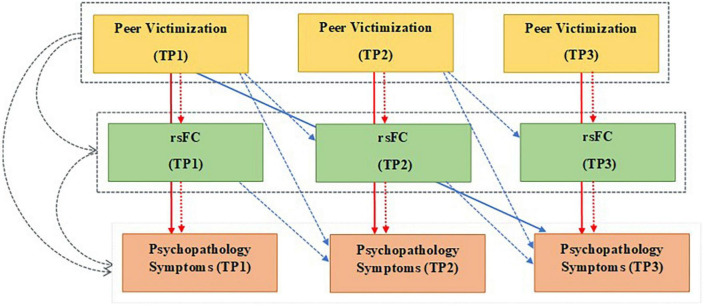
Multilevel mediation model testing the indirect effects of peer victimization on psychopathology symptoms through the resting-state functional connectivity (rsFC) using longitudinal data from three waves of the study (TP1-3). Colored arrows show three time-varying aspects of each variable: Gray: Between-person effect. Red: Within-person effect, and Blue: Lagged-within -person effect. Solid arrows: Direct effects of peer victimization on psychopathological symptoms, dashed arrows: Indirect effects of peer victimization on psychopathological symptoms through rsFC (mediators). Gray dashed boxes around peer victimization and rsFC: Average levels of each variable over three time points. TP, time point; rsFC, resting-state functional connectivity; psychopathology symptoms: internalizing and externalizing symptoms.

The present longitudinal study is also unique in that the mediational role of rsFC is investigated using an advanced multilevel linear modeling which allows to test hypotheses of temporal precedence. This analytical method provides a unique opportunity to include the parallel developmental processes, common vulnerability, and concurrency between variables, in order to evaluate the longitudinal association between variables within the same analysis (Figure 1). As several studies indicated that peer-victimized adolescents concurrently experience both internalizing and externalizing symptoms (2, 4), we included both groups of symptoms simultaneously in the mediation models. We also included a measure of childhood maltreatment (abuse and neglect) in our analyses to reduce the possible effects of earlier traumatic experiences, which might already have influenced brain connectivity and psychopathology symptoms in adolescents.

## Materials and methods

### Participants

Participants included adolescents from the Neuroventure study (26). The Neuroventure project was an imaging add-on to the larger prospective Coventure study, a cluster-randomized controlled trial evaluating the effectiveness of a brief personality-targeted substance use prevention program (27). The Coventure cohort followed annually a total of 3,966 Grade 7 students from 31 Public/Private French and English high schools in greater Montreal, Canada for 5 years. A subsample of 151 Grade 7 and 8 students (aged 12 to 14 at study baseline; 54% males) without any neurological illness was further invited to three imaging sessions over a five-year follow-up period as part of the Neuroventure study (26): Baseline (Mean age: 12.32, SD:0.65), 24-month (Mean age: 14.01, SD:0.45), and 48-month (Mean age: 17.41, SD:0.46) follow-up. Nine participants were excluded from the analyses due to incomplete data, resulting in a final sample of 142 participants (94%). Ethical approval was obtained from the CHU Sainte-Justine Research Ethics Committee and Montreal Neurological Institute and Hospital Research Ethics Board. All participants actively assented to participate while their parent consented to the study procedures.

### Behavioral measures

*Peer victimization* was measured by asking participants to retrospectively report their experiences during the past year using the validated and widely used Olweus Bullying/Victim Questionnaire—BVQ (28, 29) at each three time point. The PV subscale includes six questions that ask participants to rate their responses on a 5-point Likert scale ranging from 0 = never, 1 = only once or twice, 2 = two or three times a month, 3 = once a week, to 4 = several times a week. Responses from all items were summed to create PV total score ranging from 0 to 24. Alpha coefficient presented an acceptable reliability for the peer victimization subscale (α = 0.79).

*Internalizing Symptoms*, including symptoms of depression and anxiety, were assessed using two subscales of the Brief Symptom Inventory (BSI) (30) at each time point. Depression and anxiety subscales included a total of twelve items rated on a five-point scale (0 = not at all, 1 = a little bit, 2 = moderately, 3 = quite a bit, 4 = often). The BSI has shown a high test–retest reliability and validity (30). Responses from all items were summed to create an internalizing total score ranging from 0 to 48. Alpha coefficients highlighted good reliability for the depression subscale (α = 0.88) and for the anxiety subscale (α = 0.87).

*Externalizing Symptoms*, including symptoms of conduct and hyperactivity/inattention symptoms, were assessed using the subscales of Strengths and Difficulties Questionnaires (SDQ) (31) at each time point. The conduct and hyperactivity/inattention subscales consist of five items each rated on a three-point scale (0 = not true, 1 = sometimes true, 2 = certainly true). The SDQ is one of the most used instruments for screening psychopathology in children and adolescents. The SDQ has been widely validated in various community and clinical samples across different countries (32). Responses from all items were summed to create an externalizing total score ranging from 0 to 20. The externalizing dimension presented a good reliability (α = 0.82), and specific dimensions had acceptable reliability (ADHD dimension: α = 0.73, CD dimension: α = 0.77).

*Childhood Maltreatment* was measured using the Childhood Trauma Questionnaire (CTQ) (33) at the last follow-up. The CTQ is a 28-item self-report measure that examines retrospective experiences of childhood abuse (emotional, physical, or sexual) and neglect (physical or emotional). The scoring is based on a 5-point Likert-type scale (“never” to “frequently”). Scores on each of the CTQ domain range from 5 to 25 with the higher score presenting a greater severity of maltreatment. Responses on each of the five domains were summed for a total CTQ score. Alpha coefficients presented an acceptable reliability (α = 0.80).

### Image acquisition and preprocessing

Resting state functional magnetic resonance imaging (rsfMRI) data was collected using a 3T Siemens Magnetom Trio scanner in a single 6-min run of 152 volume of 40 axial slices with 3.5 mm isotropic voxels (TE = 30 ms, TR = 2,340 ms). While longer scan times have been shown to increase resting state reliability estimates, it is shown that the gains in inter-section reliability diminish after approximately 9–12 min in adults, and we argue that pediatric samples required even shorter scan times to reduce likelihood of motion in the scanner (34). Therefore, a 6-min scan time was considered appropriate for the population under investigation in the study. Structural images were acquired using an ultrafast gradient-echo (MPRAGE) T1-weighted sequence (192 sagittal slices, resolution 1.0 mm isotropic voxel, 256 mm FOV, TE = 2.96 ms, TR = 2,300 ms). During the resting-state sequence, participants were instructed to stay awake, remain still, and close their eyes.

Basic rsfMRI data preprocessing was carried out using FEAT (FMRI Expert Analysis Tool) Version 6.00, part of FSL (FMRIB’s Software Library)^1^ in the following order: motion correction using the MCFLIRT; slice-timing correction using Fourier-space time-series phase-shifting; non-brain removal using the BET tool; grand-mean intensity normalization of the entire 4D dataset by a single multiplicative factor. These were followed by registration to high-resolution structural and standard space images, de-obliquing and bandpass filtering between 0.1 and 0.01 Hz (35). In addition to the previous steps, detrending, nuisance regression of white matter and cerebrospinal fluid, independent component analysis (ICA) based denoising, and smoothing using a 6 mm kernel were applied on the resting state time series. Detailed description of preprocessing steps is reported elsewhere (36).

### Masking and subject level analysis

**FIGURE 2:**
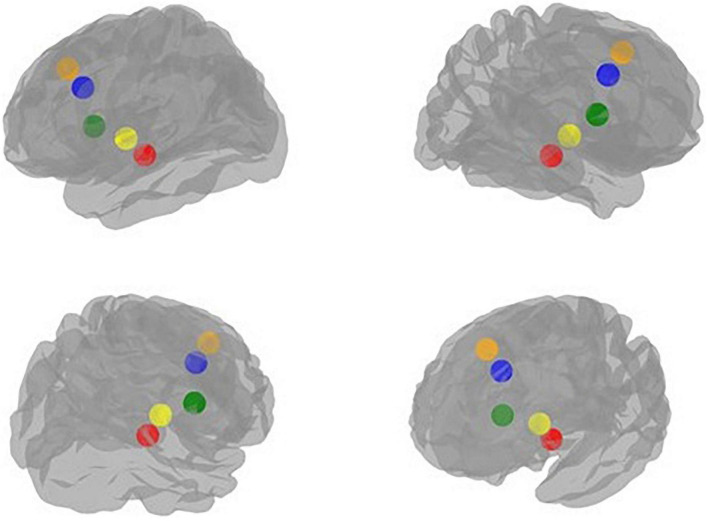
Visualization of the regions of interest (ROIs) implicated in the neural processing of emotion regulation in peer-victimized adolescents: red: Amygdala, blue: Anterior cingulate cortex (ACC), green: Anterior insula, yellow: Posterior insula, orange: Medial prefrontal cortex (mPFC).

Using the *Nilearn* module in *python* environment, for each rsfMRI scan, we computed ROI-wise connectivity maps between ROI templates extracted from a group-level functional brain atlas optimized to examine rsFC (37). Table 1 indicates details of the five regions of interest (ROIs) of this study including the amygdala, ACC, anterior and posterior insula, and mPFC (see Figure 2 for the visualization of these ROIs). We used the *Multiresolution Intrinsic Segmentation Template* (MIST) atlas to create a connectivity map using the rsfMRI data from our sample. The MIST atlas consists of functional parcellation capturing successively finer levels of spatial detail, starting with regions from seven commonly used large-scale networks: cerebellar (CER), default-mode (DMN), fronto-parietal (FPN), limbic (LIM), motor (MOT), salience (SAL), and visual (VIS). Based on the created group-level anatomical mask, participants had overlap in their amygdala and hippocampal segmentations.

**TABLE 1:** Details of the regions of interest (ROIs) most implicated in the neural processing of emotion regulation in peer-victimized adolescents.

Brain regions	Region		MNI coordinates		
Label	Size (voxel)	Symmetry	x	y	z
Amygdala	1,072	0.90	-0.31	-19.0	-14.37
Anterior cingulate cortex (ACC)	404	0.77	-0.29	20.72	28.48
Anterior insula	1,317	0.68	5.97	15.46	2.37
Posterior insula	420	0.66	-2.01	-7.26	-3.49
Medial prefrontal cortex (mPFC)	372	0.68	0.02	30.65	40.42

The current study focused on five relevant regions by the way of four specific couplings namely, amygdala-anterior insula, amygdala-posterior insula, amygdala-ACC, and amygdala-mPFC. The connectivity map was obtained by computing the z-transformed Pearson’s correlations between the average time course within each of the aforementioned ROI couplings and the time course of other ROIs in the brain.

### Group level analysis

Descriptive statistics including mean, standard deviation (SD), minimum and maximum and 95% confidence intervals (CI) for mean were calculated for total scores of PV and psychopathology (internalizing and externalizing) symptoms at each assessment time point.

Multilevel mediation analysis was utilized using MPLUS 8.4 software to test the indirect effects of PV on internalizing/externalizing symptoms through rsFC using separate models for each coupling (i.e., four models in total). Based on person mean centering, three aspects of the predictor and mediator variables were distinguished: between-person, concurrent within-person, and lagged within-person effects. As an example, the predictor variable, PV, was operationalized into a between-person effect (average level of reported PV over three time points), a concurrent within-person effect (current change in the level of reported PV compared to the person’s mean PV level), and a lagged within-person effect (change in the level of reported PV at the last assessment compared to the person’s mean PV level on a given dependent variable; see Figure 1). Three levels of the mediator variables (rsFC couplings) were similarly operationalized. Considering the correlation between outcome variables (internalizing and externalizing symptoms), a multivariate approach was used to model the temporal mediation for both outcomes simultaneously. Regarding the covariates, all models controlled for the fixed effects of self-reported childhood maltreatment assessed in third wave of the study and baseline sex (male vs. female), and linear effect of the time parameter (coded from one to three based on the study waves). Including other associations besides the mediation effect in the model will allow us to test for their concurrent effect on each other and exclude their possible effects on the outcomes. As separate models were estimated for four different set of mediators (i.e., coupling), and to control for the inflated chance of type II error due to multiple testing, we used the False Discovery Rate procedure proposed by Benjamini with a *q*-value of 0.05 (38).

## Results

Table 2 presents descriptive statistics of peer victimization and internalizing and externalizing symptoms at each assessment time point of the study. PV and externalizing symptoms were slightly decreased over the three time points of study, whereas, internalizing symptoms were slightly increased.

**TABLE 2:** Descriptive statistics of peer victimization and internalizing and externalizing symptoms at each assessment time point.

	Mean	SD	Minimum	Maximum	95% CI for mean
***Peer victimization***					
Time point 1	3.14	2.79	1	12	1–10
Time point 2	2.45	2.01	1	9	1–7.5
Time point 3	2.35	2.20	1	10	1–8
***Internalizing symptoms***					
Time point 1	7.59	7.93	0	44	0–28.05
Time point 2	7.63	8.21	0	48	0–28
Time point 3	9.90	9.61	0	48	0–42.25
***Externalizing symptoms***					
Time point 1	6.27	3.57	0	19	0.48–14
Time point 2	6.29	3.57	0	17	1–14.48
Time point 3	5.75	3.36	0	14	1–13.63

**FIGURE 3:**
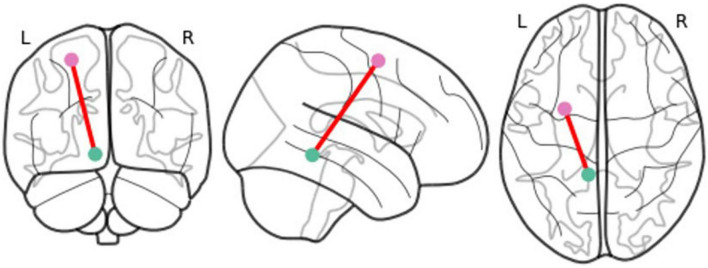
Visualization of the coupling which significantly mediated the relationship between peer victimization and lagged within-person internalizing symptoms: Amygdala-ventral posterior insula resting-state functional connectivity.

Results of the multivariate longitudinal mediation models indicated that, increased rsFC of the amygdala-ventral posterior insula mediated the lagged within-person effect of PV on internalizing symptoms (β = 0.144; *P* = 0.036; 95% CI [0.018,0.332]) (see Table 3 and Figure 3). This effect was significant regardless of childhood maltreatment, sex differences, and concurrent externalizing (conduct and hyperactivity/inattention) symptoms. None of the couplings of amygdala-anterior insula, amygdala-ACC and amygdala-mPFC significantly mediated the relationship between PV and psychopathology (internalizing and externalizing) symptoms. In addition, no significant mediation effect for rsFC was found in the relationship between PV and externalizing symptoms.

**TABLE 3:** Indirect effects of peer victimization on internalizing and externalizing symptoms through resting-state functional connectivity.

Mediating couplings	Internalizing symptoms		Externalizing symptoms	
	β	SE	β	SE
***Amygdala & anterior insula mediating coupling***				
Within-person	0.022	0.033	0.008	0.013
Between-person	0.037	0.920	-0.048	0.539
Lagged within-person	0.053	0.053	0.005	0.015
***Amygdala & posterior insula mediating coupling***				
Within-person	0.060	0.049	0.005	0.012
Between-person	0.250	13.905	0.018	5.541
Lagged within-person	0.144*	0.081	0.009	0.022
***Amygdala & mPFC mediating coupling***				
Within-person	0.002	0.021	-0.001	0.007
Between-person	-0.121	4.239	0.004	1.830
Lagged within-person	0.001	0.026	0.003	0.012
***Amygdala & ACC mediating coupling***				
Within-person	0.008	0.027	0.001	0.008
Between-person	-0.052	2.606	-0.109	1.505
Lagged within-person	0.001	0.027	0.001	0.009

mPFC, medial prefrontal cortex; ACC, anterior cingulate cortex.

*P < 0.05.

## Discussion

This study aimed to identify the mediating effects of the amygdala rsFC with ACC, anterior and posterior insula, and mPFC in the relationship between peer victimization and subsequent psychopathology (internalizing and externalizing) symptoms over a period of five years in adolescence. Findings from this study are in line with literature indicating PV adversely affects neural response in adolescence and through that increases the risk of psychopathology (6, 12, 39). Our findings extend this literature by indicating that exposure to PV is related to delayed emergence of internalizing (anxiety and depression) symptoms in late adolescence indirectly through rsFC alterations in the amygdala-ventral posterior insula. Notably, these findings were independent of other types of traumatic experiences (i.e., childhood maltreatment), sex differences, and concurrent externalizing (conduct and hyperactivity/inattention) symptoms. To our knowledge, our findings are the first to provide new insights into a possible rsFC marker explaining the emergence of internalizing symptoms in peer-victimized youth in late adolescence.

Posterior insula is generally implicated in the somatosensory processing of pain, such as recognition, coding of intensity and location, and memory of physical pain (40–42). Some studies have shown that activity in posterior insula also enhances in response to intense experiences of social pain and exclusion, such as romantic rejection (43, 44). Posterior insula has also been implicated in intrusive or “re-experiencing” symptoms of post-traumatic stress disorders (PTSD) (e.g., flashbacks, nightmares, recurrent and unwanted distressing memories) (45, 46). Exposure to PV during adolescence is also an intense and traumatic experience for many youth which impacts their self-esteem and threatens their feelings of social connection (47, 48). Increased connectivity of the amygdala and the posterior insula at rest could indicate a long-lasting maladaptive communication between emotional response and sensory perception of pain (i.e., bottom-up emotion regulation) in peer-victimized adolescents which makes them vulnerable to internalizing symptoms in late adolescence. Increased rsFC between amygdala and posterior insula has also been reported in patients with PTSD (49). Other studies have shown that enhanced functional connectivity between amygdala and posterior insula is associated with habituation to negative stimuli (50). Habituation is an adaptive response to repeated exposure to negative or aversive stimuli and helps individuals to survive in the context of adversity (50, 51). Theories on childhood maltreatment have indicated that such neuroplastic adaptive responses may help the maltreated child to survive in an uncertain and threatening environment (e.g., see a review paper by (52). Habituation may help adolescents to decrease their physiological responses to victimization experiences in the short term but result to psychopathology in the long term. In this line, McCurry et al. (53) recently indicated that patients with more severe re-experiencing symptoms of PTSD show increased habituation when viewing negative compared with neutral images in brain regions involved in suppression of emotions and thoughts (i.e., functional connectivity of amygdala with left inferior frontal gyrus and dorsal ACC). This process may result in subsequent rebound of intrusive and unwanted thoughts in these individuals (53). Similarly, adolescents exposed to PV may adapt by suppressing negative emotions associated with the experience of victimization (e.g., shame, sadness, loneliness, threat) to numb or dissociate themselves from the social pain in short term. However, this may lead to alterations in networks involved in the bottom-up emotional regulation (i.e., amygdala-posterior insula connectivity) and reexperiencing of these negative emotions and memories, which in long term, increases the risk of internalizing (anxiety and depression) symptoms in peer-victimized adolescents.

We did not find any significant mediating effects for the rsFC of amygdala-ACC and amygdala-mPFC between PV and internalizing symptoms. The study by McIver et al. (12) indicated that positive functional connectivity of the mPFC-left amygdala during a social inclusion/exclusion task moderates the association between PV and depressive symptoms in adolescents. The contrast between this finding and findings from our study might be explained by our different imaging approach (task vs. rest) and statistical analyses (moderation and cross-sectional vs. mediation and longitudinal). In this line, results from studies of amygdala coupling with other brain regions during adolescence indicated that strength and direction of these couplings are highly influenced as a function of task demands (54). It is then possible that the amygdala co-activates with the ACC and mPFC in task-based studies. Our findings suggest that the long-term effects of PV on adolescent internalizing symptoms is more prominent through alterations in rsFC of regions underlying bottom-up emotion regulation of emotion rather than the top-down regulation.

These findings have important clinical implications for reducing the risk of internalizing problems in adolescents exposed to PV. While interventions, such as cognitive reappraisal, have been used to reinterpret an emotion-eliciting situation with the aim of decreasing negative thoughts and emotions in people with internalizing problems, a bottom-up somatic approach, such as hypothesized in the Trauma Resiliency Model (TRM) which, focuses on skills that use sensory awareness for emotion regulation and integration (55), may be a better approach for reducing internalizing symptoms in adolescents exposed to PV.

No significant mediation effect was found for the amygdala rsFC with ACC, insula and mPFC in the relationship between PV and externalizing (conduct and hyperactivity/inattention) symptoms. We did not find any study that examined the functional connectivity (neither task-based, nor resting-state) of these regions in the association between PV and adolescent’s vulnerability to externalizing symptoms. Therefore, it is difficult to interpret our lack of significant rsFC mediation effects between PV and externalizing symptoms. One reason that we did not see any significant effects for externalizing symptoms might be that the impact of PV on areas associated with emotional dysregulation does not manifest as changes in rsFC but appears as altered neural response to explicit tasks. For example, a fMRI study, which assessed neural responses to risk-taking tasks in peer-victimized adolescents, indicated that heightened activities in bilateral amygdala, dorsomedial PFC (dmPFC), and posterior superior temporal sulcus (pSTS) in response to such tasks mediate the association between chronic PV and self-reported antisocial behaviors in daily life (16). Future studies are required to compare different methodological and analytical approaches in relation to the emergence of externalizing symptoms in adolescents exposed to PV.

Our results should be considered in light of several limitations. First, we used self-report measures for assessing PV and psychopathology symptoms and our assessment did not involve more objective methods of measuring these behaviors, such as nominating by peers for PV and/or structured or semi-structured interviews with clinicians for assessing symptoms. Second, we did not assess important characteristics of the PV, such as severity, duration, or age of occurrence, each of which may affect the outcomes. It is possible that participants had been exposed to peer victimization before entering this study and that affected their rsFC of the studied regions. Third, although we accounted for factors that may influence our outcomes, it is possible that other variables, such as individual (e.g., substance use, puberty), and/or environmental and systemic (e.g., poverty, peer group, parenting) factors also affected the outcome behaviors. Additional research is needed to shed more light into these interactions. Finally, our sample size was relatively small considering the complexity of the model used to analyze the data. We recommend that this model be tested again using longitudinal data with larger sample sizes, such as the IMAGEN study (56).

Despite these limitations, the present study is one of the first to indicate that peer-victimized adolescents differentially process the experience of victimization at the rsFC level and that these neural patterns may serve as a unique marker of persistent internalizing symptoms. These findings further highlight the impact of interpersonal difficulties with peers on adolescent neural response involved in regulation of emotions, and its effect on development of psychopathology. Early interventions targeting emotion regulation, physiological response to negative emotions, and social perception may reduce the impact of PV on neurological alterations underlying the development of internalizing problems in peer-victimized adolescents.

## Data availability statement

The raw data supporting the conclusions of this article will be made available by the authors, without undue reservation.

## Ethics statement

The studies involving human participants were reviewed and approved by CHU Sainte-Justine Research Ethics Committee and Montreal Neurological Institute and Hospital Research Ethics Board. Written informed consent to participate in this study was provided by the participants’ legal guardian/next of kin.

## Author contributions

HE designed the concept of the original study and wrote the first draft. MA led the statistical analysis. SS, HE, and JB contributed to the preparation of data and their analyses. PC and AD supervised the original Neuroventure project. All authors interpreted the results and critically revised the manuscript for important intellectual content.

## Footnotes

1
www.fmrib.ox.ac.uk/fsl
